# Fabrication of Nanoparticle/Polymer Composite Photocatalytic Membrane for Domestic Sewage In Situ Treatment

**DOI:** 10.3390/ma15072466

**Published:** 2022-03-27

**Authors:** Yawei Yang, Tao Wu, Wenxiu Que

**Affiliations:** Electronic Materials Research Laboratory, Key Laboratory of the Ministry of Education, International Center for Dielectric Research, Shaanxi Engineering Research Center of Advanced Energy Materials and Devices, School of Electronic Science and Engineering, Xi’an Jiaotong University, Xi’an 710049, China; ttaowu@stu.xjtu.edu.cn

**Keywords:** nanoparticle, polymer, photocatalysis, membrane

## Abstract

Photocatalytic technology using semiconductor catalysts is a promising candidate for light-polluted water treatment. In the past decades, TiO_2_-related nanomaterials and photocatalytic devices have been applied for sewage ex-situ treatment. However, in situ photocatalytic technology using functional membranes is still needed for many large-scale outdoor scenarios. This work successfully fabricated a robust reusable photocatalytic membrane by firmly immobilizing TiO_2_ nanoparticles on polymer membranes, supported by various plastic substrates, through an industrial membrane blowing process. The as-fabricated photocatalytic membrane was fabricated by all low-cost and eco-friendly commercial materials and exhibited stable photocatalytic performance in domestic sewage in situ treatment in natural conditions. This work is expected to promote the photocatalytic membrane for practical application.

## 1. Introduction

Advanced oxidation processes (AOPs), including photochemical, phonochemical, ozonic, electrochemical, and Fenton oxidations, which yield highly reactive oxygen species (ROS) for decomposing organics, offer the compelling advantage of ideally destroying organic pollutants instead of simply diverting them from the water [1]. In photocatalysis, one of the most promising AOPs involves semiconductor catalysts with appropriate band energy levels that generate electrons and holes by absorbing and converting photons. Then, the ROS are produced through charge carrier transfer to profoundly degrade the organic matter in water. They are suitable for light-polluted water, such as water sources, rivers, biologically pretreated wastewater, and water ponds with unpleasant odors [2] and are ideal complements to microbial degradation technology.

Many narrow bandgap semiconductor photocatalysts with wide solar spectrum response, such as ZnO, metal sulfides, and carbon nitrides, have been developed for photocatalytic degradation of simulated organic pollutants [3,4,5]. Currently, the TiO_2_ photocatalyst has been well studied and is the most practical semiconductor photocatalyst for real wastewater treatment due to its high stability and non-toxic properties [2]. However, catalysts in a nanopowder form cannot be quickly recovered and always become a secondary contamination, and therefore they cannot be directly applied to sewage treatment [2]. In the past decades, TiO_2_ nanoparticles have been fixed in various photocatalytic devices, in which sewage is supplied by external force, to degrade the organic pollutants under UV lamp [1,6,7]. Although these ex-situ photocatalytic technologies have been well established, the in situ ones are necessary for many large-scale outdoor application scenarios, such as domestic sewage, river, and water source treatment. Generally, membrane-type photocatalysts meet the requirement of in situ sewage treatment where the catalyst powder is contained [8]. In the practical application, the photocatalytic membrane is put on the water surface to accept sunlight and fully contact sewage and air (i.e., oxygen), meanwhile, avoiding the secondary pollution of nanopowder dispersion in water [9].

Recently, typical photocatalytic membranes have been fabricated by attaching catalysts to polymer fiber or metal mesh substrates through electrospinning, spraying, and other processes using polymer as a binder, such as polyacrylonitrile (PAN), polyvinylidene fluoride (PVDF), polystyrene (PS), polymethyl methacrylate (PMMA), etc. [10,11,12,13,14]. However, they are usually invalid for catalysts leaching from the substrates [15]. In addition, there are very few photocatalytic membrane manufacturers in the world, and therefore it continues to be challenging to design an elegant system for the practical application of these photocatalytic membranes [16].

Herein, to respond to the stability and recovery issue for practical domestic sewage in situ treatment, a robust reusable photocatalytic membrane is reported in this work. An industrial membrane blowing process, which is widely applied for continuous polymer/plastic membrane production in industry, was developed for photocatalytic membrane fabrication by immobilizing TiO_2_ nanoparticles on a polymer membrane supported by a plastic substrate. It is hypothesized that this facile, low-cost, and eco-friendly photocatalytic membrane would be effective for large-scale outdoor sewage in situ treatment.

## 2. Materials and Methods

### 2.1. Materials

TiO_2_ (P25) and waterborne polyurethane (PU, 60% solid content) were purchased from Macklin, Shanghai, China. PVDF (HSV900) was purchased from Kynar, France. N,N-dimethylformamide (DMF, 99.0%) and ethyl acetate (99.5%) were purchased from Sinopharm, Shanghai, China. The polyethylene terephthalate (PET) cloth was provided by Foshan Textile Mill, Foshan, China. All chemicals were used as received without further purification.

### 2.2. Fabrication of the Photocatalytic Membranes

For the filled photocatalytic membrane (FPM), 30 g TiO_2_, 3 g PVDF, and 100 g PU were mixed into 20 g DMF and 70 g ethyl acetate solution. The FPM was obtained by a modified industrial membrane blowing process (Figure S1), which fit the P25 + PU mixture onto 1 m^2^ of PET substrate to form a continuous membrane. Finally, the membrane was dried at 130 °C for 20 min (Figure S2).

For the bonded photocatalytic membrane (BPM) (traditional way of photocatalytic membrane preparation and for comparison), 3 g PVDF was dissolved in 600 g DMF under stirring. Then, 30 g P25 was put into the PVDF solution to form a uniform slurry. The FBM was obtained by a padding process (use the same machine as shown in Figure S1), which coated the slurry onto the 1 m^2^ PET fiber cloth. Finally, the membrane was dried at 135 °C for 20 min (Figure S3).

### 2.3. Characterizations

The morphology and elemental analysis of the samples were observed by a field emission scanning electron microscopy equipped with an energy dispersive X-ray spectroscopy (SEM-EDS, Quatan 250FEG, FEI, Hillsboro, OR, USA).

### 2.4. Photocatalytic Measurements

For the simulated sewage treatment, a 300 W Xe lamp (CEL-HXF300, Au-light, Beijing, China) equipped with an AM 1.5G filter (1 kW/m^2^) was used as light source. A piece of circular photocatalytic membrane with a diameter of 4 cm was put into 120 mL methyl orange (MO) or tetracycline hydrochloride (Tch) aqueous solution (10 mg/L). The distance from the photocatalytic membrane to the water surface was 0.5 cm. The reaction system was kept at 6 °C (Figure S4). The concentration of the residual MO or Tch was monitored at a sequence of time intervals by the UV-Vis spectrum to calculate the degradation rate based on the Beer–Lambert Law. N_2_, ammonium oxalate (AO), benzoquinone (BQ), and *tert*-butyl alcohol (TBA) were applied as O_2_, h^+^, O_2_^−^•, and OH• sacrificial agents, respectively, to identify the active species. In addition, two commercially available TiO_2_ photocatalytic membranes (named C1 and C2, Figure S5) were also compared.

For the domestic sewage treatment, a piece of the rectangular photocatalytic membrane was floated on the domestic sewage (Figure S6) in a box under natural sunlight. The sewage was replenished every two days. The contamination indexes, chemical oxygen demand (COD), NH_3_-N, total nitrogen (TN), and total phosphorus (TP) were measured by a water quality detector (LH-T725, Lohand, China). The pH value was measured by a pH meter.

## 3. Results and Discussion

**Figure 1:**
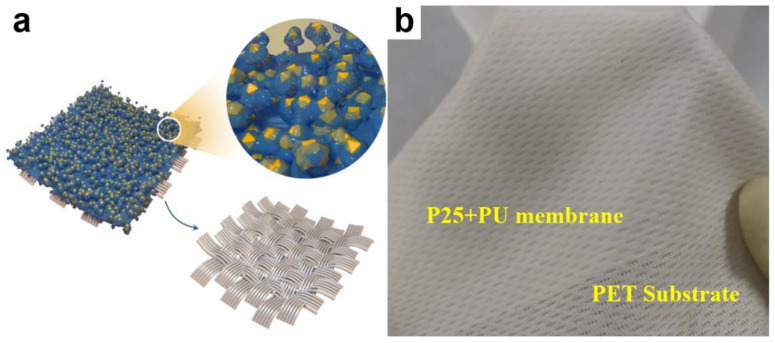

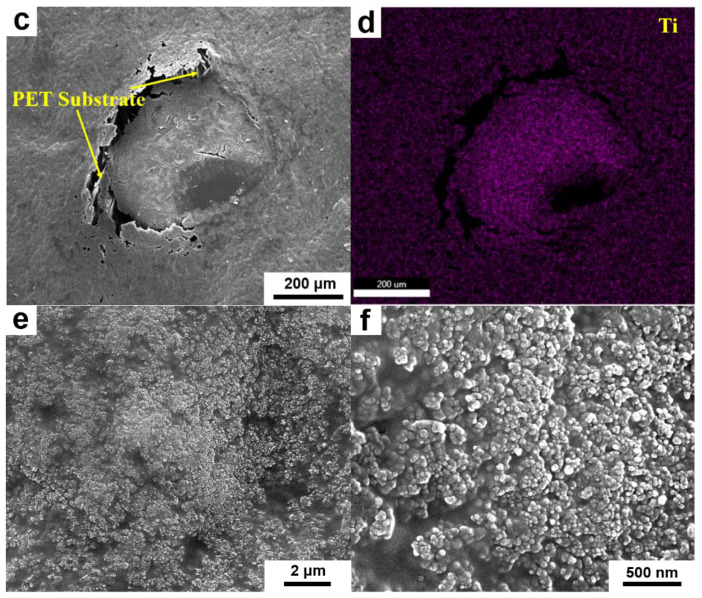
(**a**) Scheme; (**b**) photo; (**c**,**d**) low-magnification SEM image and corresponding EDS mapping of Ti element (**e**,**f**) high-magnification SEM images of the FPM.

By the membrane blowing process, a consecutively smooth PU membrane can fit the PET fiber fabric substrate (Figure S7a,b), which is the precondition of the FPM fabrication. The PET substrate has the advantage of being physically and chemically stable in water. The P25 + PU membrane can be fit well in various PET substrates on a large scale, including fiber fabric and gauze (Figure 1a,b and Figure S2). The P25 nanoparticles inserted in PU make the membrane rougher (Figure 1c and Figure S7a). The P25 nanoparticles are filled fully, uniformly, and tightly on the PU membrane surface, maintaining their original particle sizes (Figure 1d–f). However, there is a slight aggregation and cover of the nanoparticles by PU (Figure 1f) at a cost of the stability of the membrane. There is a difference between the FPM and BPM. For the FPM, the PET substrate acts as the holder for supporting the PU membrane (Figure 1c and Figure S7c), in which the nanoparticles are immersed. For the BPM, the P25 nanoparticles are bonded onto the PET fibers by PVDF binder. The P25 nanoparticles are aggregated and distributed more randomly on the fibers, whereas less nanoparticles are blocked by the polymer (Figure S3). This indicates that more P25 nanoparticles can participate in the photocatalytic reaction than the FPM.

**Figure 2:**
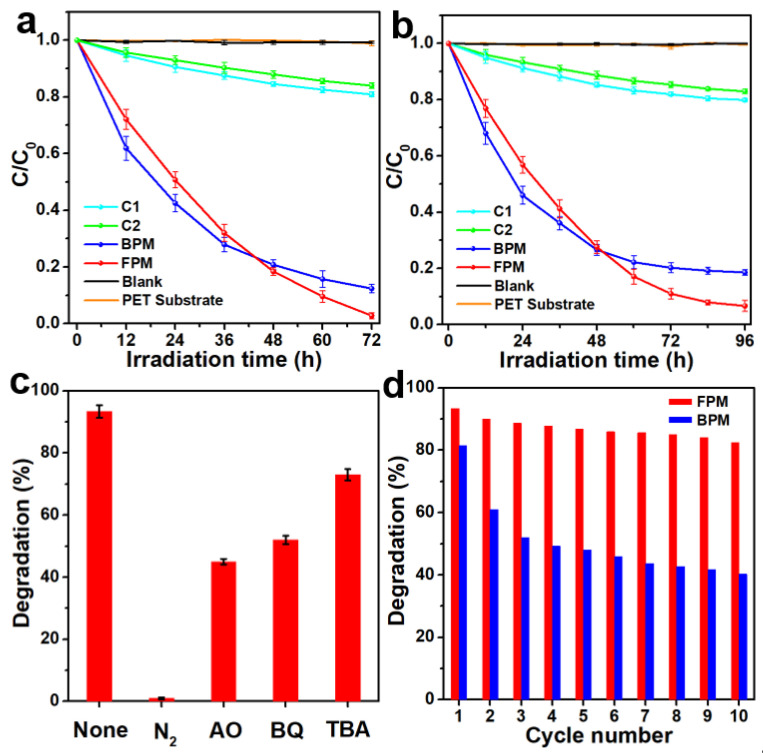
Photocatalytic performance: (**a**) MO degradation; (**b**) Tch degradation; (**c**) Tch degradation of the FPM under different kinds of ROS sacrificial agents; (**d**) cycle test of Tch degradation with each cycle for four days.

Photocatalytic activities of the FPM and BPM were evaluated by MO dye and colorless Tch degradation (Figure S4). Commercially available photocatalytic membranes C1 and C2 were used for comparison. There was no degradation observed in the blank testing and for the pure PET substrate, indicating the stability of the simulated sewage. For MO degradation (Figure 2a), only 19% and 16% MO were photodegraded by using C1 and C2 after three-day irradiation. In comparison, 97% and 88% were photodegraded using the FPM and BPM, respectively, showing significantly enhanced photocatalytic activities. The photodegradation rate of the BPM is faster than that of the FPM at the early stage, but it slows down after 48 h, which can be attributed to the powder separating gradually from the PET fibers (Figure S8). The same trend can be seen in Tch degradation (Figure 2b), 20%, 17%, 93%, and 81% Tch are photodegraded by using C1, C2, the FPM, and the BPM, respectively, after four-day irradiation. These results suggest that the FPM is promising for sewage in situ treatment.

To identify the active species for the FPM, ROS sacrificial agents were added to the reaction system. A significant decrease in Tch degradation occurs in the absence of O_2_ and in the presence of h^+^, O_2_^−^•, and OH• scavengers (Figure 2c), indicating that all h^+^, O_2_^−^•, and OH• are actively participating in the photocatalytic process, the same as TiO_2_ powder photocatalysts [17]. The photocatalytic activity of the BPM gradually decreases over 10 cycles due to the powder loss. On the contrary, the FPM can maintain its high photocatalytic activity for at least forty days without nanoparticle loss (Figure 2d). Although, initially, the photocatalytic activity of the BPM is better than that of the FPM, due to more exposure of nanoparticles, the FPM is more stable, and is reusable for practical sewage in situ treatment.

**Figure 3:**
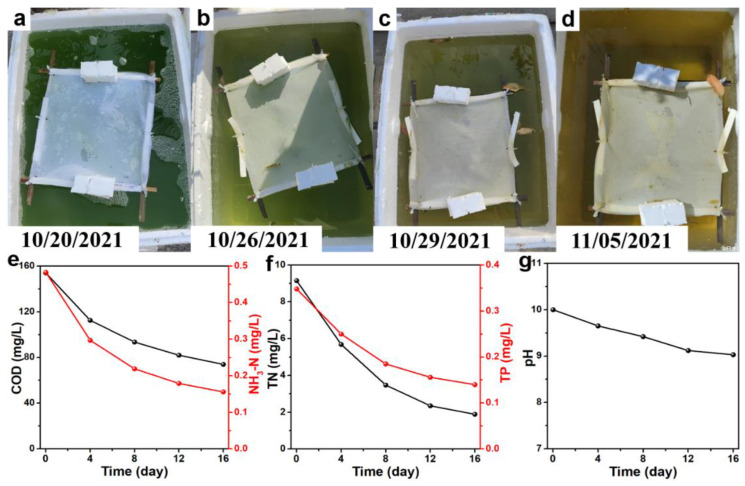
(**a**–**d**) Photos of photocatalytic processes of the FPM in autumn (20 October~5 November 2021); photocatalytic performance of the FPM for domestic sewage treatment: (**e**) COD and NH_3_-N removal; (**f**) TN and TP removal; (**g**) pH value.

The FPM was applied for domestic sewage in situ treatment. The feculent green sewage turns to clear bottom, and all contamination indexes are significantly reduced after one week in summer (Figure S9 and Table S1). It was also applied in autumn when both the light intensity and temperature were decreased. As expected, it takes a longer time for photocatalytic degradation. The green alga is killed over half a month, making the water clear (Figure 3a–d). The COD, NH_3_-N, TN, TP, and pH values are obviously reduced from 154 to 74 mg/L, from 0.483 to 0.156 mg/L, from 9.15 to 1.89 mg/L, from 0.348 to 0.140 mg/L, and from 10.00 to 9.03, respectively (Figure 3e–g). The treated water no longer has an unpleasant odor. After sewage treatment, the immobilization of nanoparticles in FPM is much better than in BPM (Figure S10).

## 4. Conclusions

This study developed a modified industrial membrane blowing process for photocatalytic membrane fabrication. A robust reusable photocatalytic membrane was successfully fabricated by immobilizing catalyst nanoparticles on a PU membrane, supported by a PET substrate. The as-fabricated FPM exhibited satisfactory recycling ability and photocatalytic activity for domestic sewage in situ treatment in natural conditions. The present study is expected to provide a new approach for promoting the photocatalytic membrane for practical application.

## Data Availability

The data presented in this study are available on request from the corresponding author.

## Supplementary Materials

The following supporting information can be downloaded at: https://www.mdpi.com/article/10.3390/ma15072466/s1, Figure S1. Photo of an industrial membrane blowing machine for membrane blowing and padding processes, Figure S2. Photo of a large-scale filled photocatalytic membrane (FPM) on different kinds of PET substrates (fiber fabric and gauze), Figure S3. (a) Photo and (b,c) corresponding SEM images of the bonded photocatalytic membrane (BPM), Figure S4. (a–c) Photocatalytic MO degradation processes of the FPM; and (d) Photocatalytic MO degradation results after 72 h, Figure S5. Photos of the commercially available TiO2 photocatalytic membranes: (a) sweater-type membrane (C1), and (b) paper-type membrane (C2), Figure S6. Photo of domestic sewage source (Quanzhou, Fujian Province, China), Figure S7. SEM images of the (a) pure PU membrane, (b) PET substrate (fiber fabric), and (c) the artificially cracked FPM, showing PET fiber substrate supported P25+PU membrane, Figure S8. Photo of powder separated from the BPM in water, Figure S9. (a–c) Photos of photocatalytic processes of the FPM in summer (28 July~4 August., 2021), Figure S10. SEM images of the photocatalytic membranes (a,b) the FPM (c,d) the BPM after domestic sewage treatment, Table S1. Photocatalytic performance of the FPM for outdoor sewage treatment in Summer.
