# Supplementary material for: Fabrication of Nanoparticle/Polymer Composite Photocatalytic Membrane for Domestic Sewage In Situ Treatment
Source: Materials (Basel). 2022 Mar 27;15(7):2466. doi: 10.3390/ma15072466 (PMC8999259; doi:10.3390/ma15072466)
Supplement: Supplementary file 1 [file materials-15-02466-s001.zip › materials-1653840-supplementary.pdf]

Supporting Information

Fabrication of Nanoparticle/Polymer Composite Photocatalytic Membrane for Domestic Sewage In Situ Treatment

Yawei Yang *, Tao Wu, Wenxiu Que *

Electronic Materials Research Laboratory, Key Laboratory of the Ministry of Education, International Center for Dielectric Research, and Shaanxi Engineering Research Center of Advanced Energy Materials and Devices, School of Electronic Science and Engineering, Xi'an Jiaotong University, Xi'an 710049, P. R. China

*Corresponding authors:
Tel. & Fax: +86-29-83395679
Email: ywyang@xjtu.edu.cn (Y. Yang) wxque@xjtu.edu.cn (W. Que)

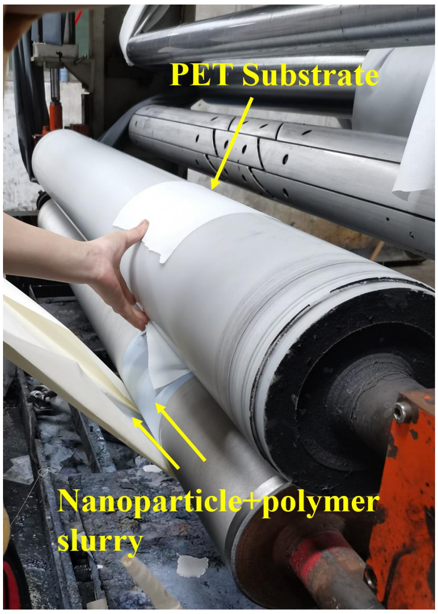

Figure S1. Photo of an industrial membrane blowing machine for membrane blowing and padding processes.

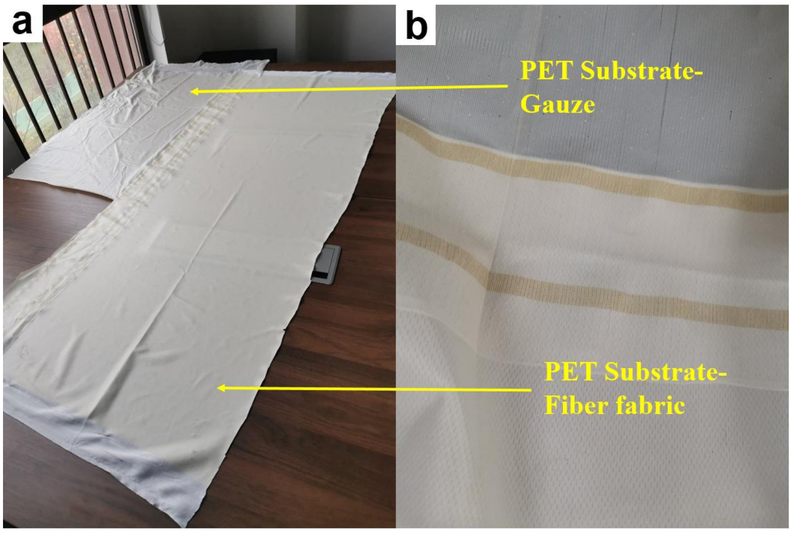

Figure S2. Photo of a large-scale filled photocatalytic membrane (FPM) on different kinds of PET substrates (fiber fabric and gauze).

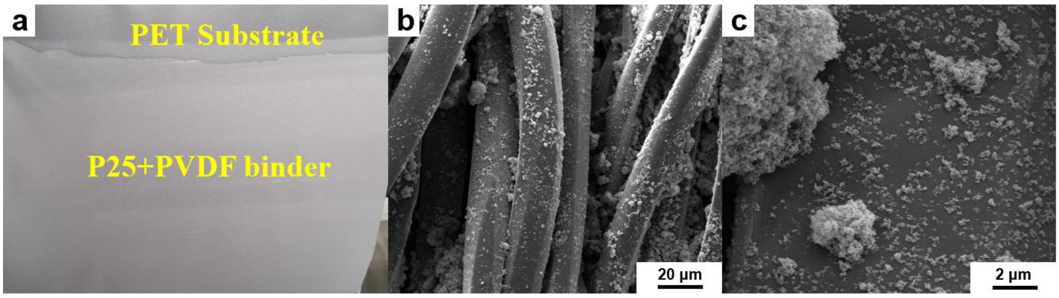

Figure S3. (a) Photo and (b, c) corresponding SEM images of the bonded photocatalytic membrane (BPM).

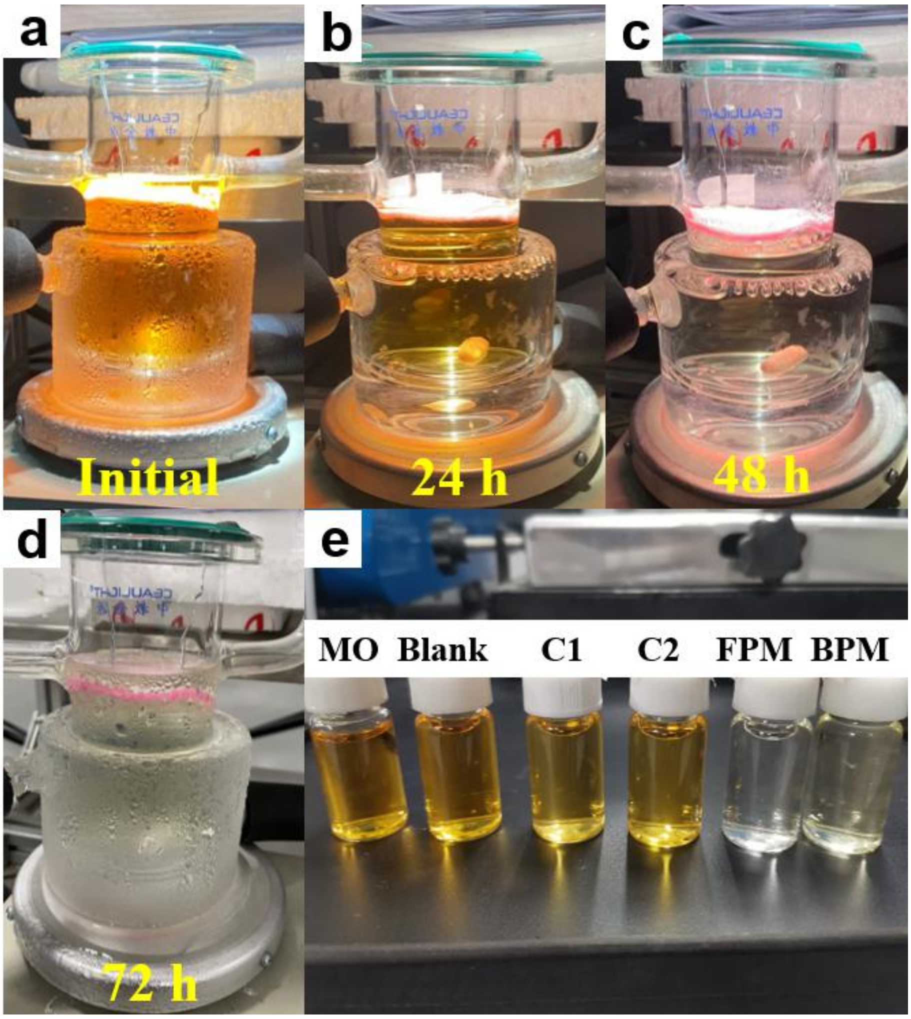

Figure S4. (a-c) Photocatalytic MO degradation processes of the FPM; and (d) Photocatalytic MO degradation results after 72 h.

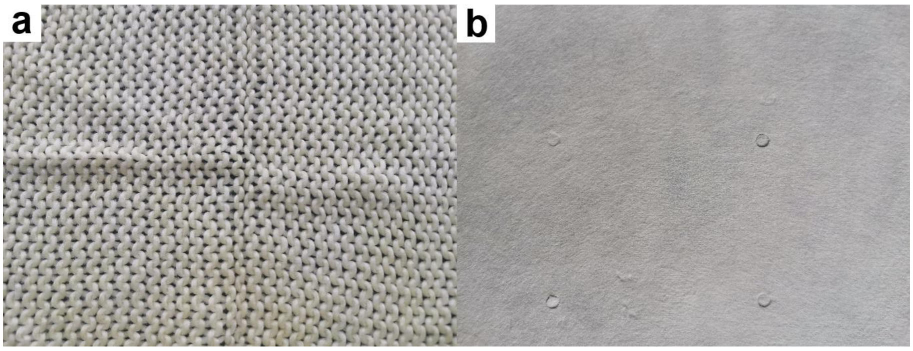

Figure S5. Photos of the commercially available TiO_2 photocatalytic membranes: (a) sweater-type membrane (C1), and (b) paper-type membrane (C2).

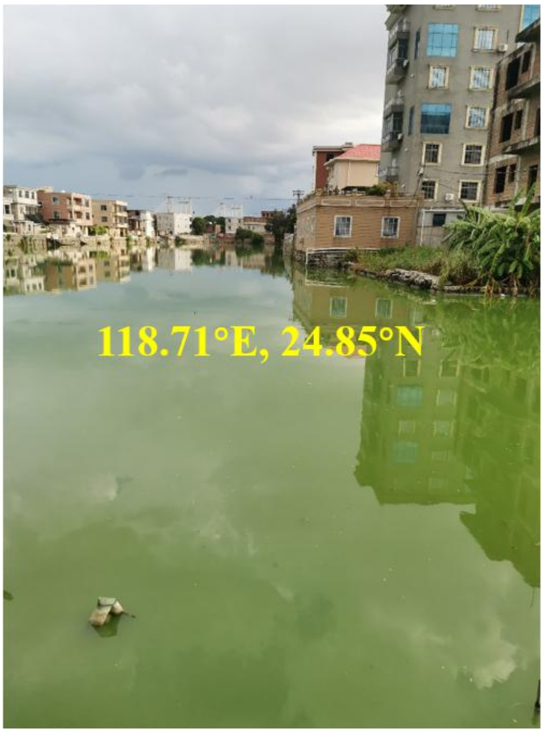

Figure S6. Photo of domestic sewage source (Quanzhou, Fujian Province, China).

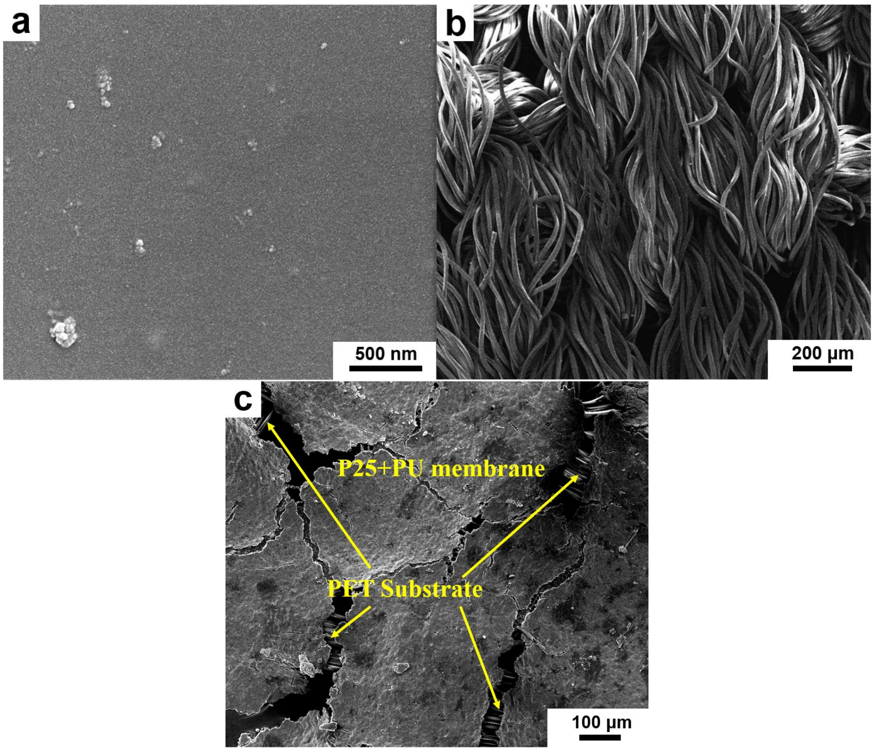

Figure S7. SEM images of the (a) pure PU membrane, (b) PET substrate (fiber fabric), and (c) the artificially cracked FPM, showing PET fiber substrate supported P25+PU membrane.

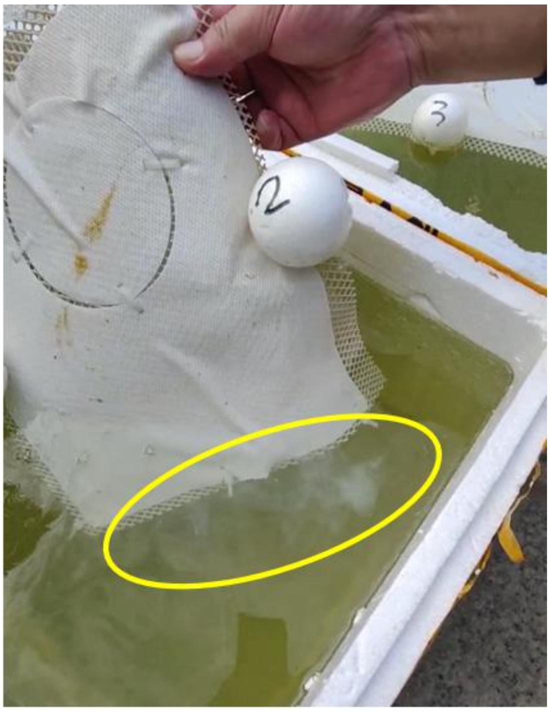

Figure S8. Photo of powder separated from the BPM in water.

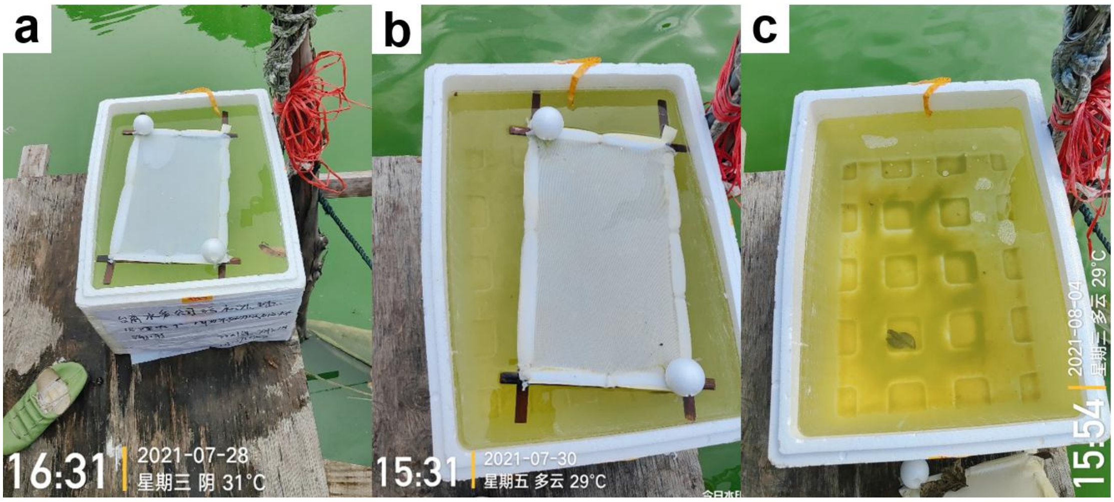

Figure S9. (a-c) Photos of photocatalytic processes of the FPM in summer (28 July~4 August, 2021).

Table S1. Photocatalytic performance of the FPM for outdoor sewage treatment in Summer.

	28 July, 2021	4 August, 2021
COD	94	41
NH_3-N	0.75	0.26
TN	3.65	1.83
TP	0.37	0.01
pH value	10.05	9.02

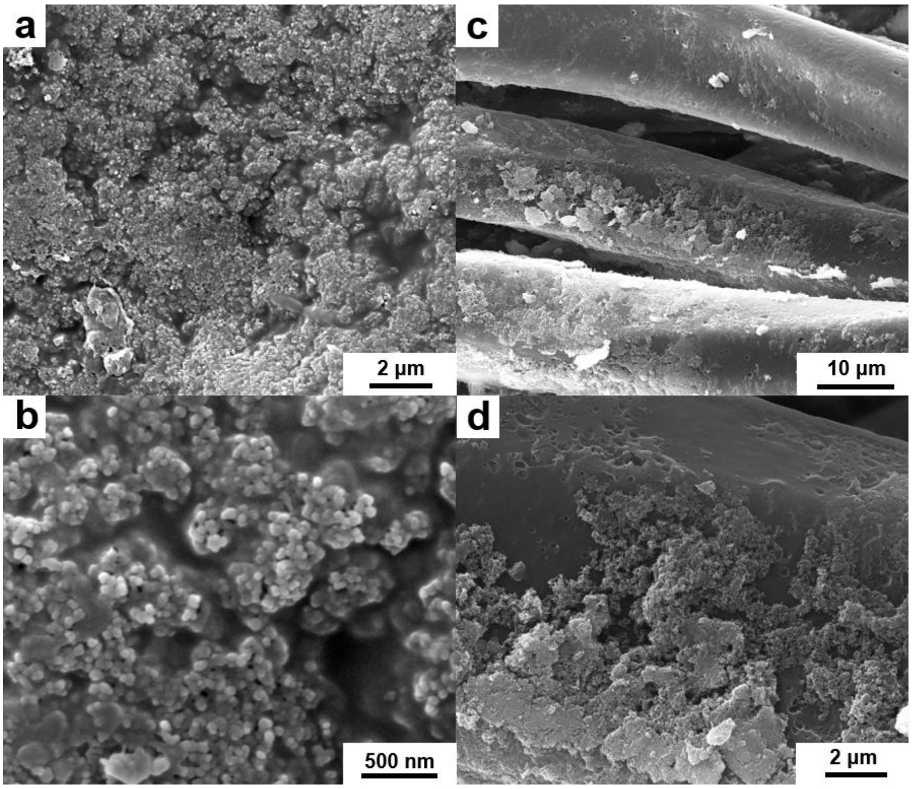

Figure S10. SEM images of the photocatalytic membranes (a, b) the FPM (c, d) the BPM after domestic sewage treatment.
